# Mr. W. J. Ansorge

**Published:** 1913-11-15

**Authors:** 


### An Interesting Career.

The death of Mr. W. J. Ansorge removes a member of the medical profession whose career and many-sided activities were distinctly out of the ordinary. He was the son of a missionary, was born in India in 1850, and was brought up in Mauritius. After graduating in Arts at Cambridge, Mr. Ansorge returned to Mauritius, where he was appointed in 1872 a professor at the Royal College, at the early age of twenty-two. Here he remained until 1886, having been senior professor from 1880 onwards. Next he determined to enter the medical profession, and proceeded to St. Bartholomew's for the purpose. Here he won a Jeaffreson exhibition in 1887, and he qualified as M.R.C.S., M.R.C.P., in 1892. He entered the Uganda Medical Service, and at the time of the Soudanese mutiny in 1897 was stationed at Masindi in Unyoro, one of the furthest outposts of civilisation in those days. While General Macdonald was taking the necessary steps to quell the insurrection a rumour came in that the Unyoro troops had also mutinied and had murdered all their officers, including Mr. Ansorge. This turned out not to be true: the soldiers had been tampered with, but had remained loyal. Soon afterwards Mr. Ansorge brought the Masindi garrison to Kampala, where they were disarmed as a precaution.

Throughout his service in Uganda Mr. Ansorge took advantage of his opportunities for zoological research; on birds and insects especially he became an authority of the first rank. In 1899 he published an important book entitled "Under the African Sun," containing valuable accounts of his adventures and his zoological researches. He was by this time nearly fifty years of age, and possessed a long white beard, which gave him a most patriarchal appearance. In 1900 he travelled right across Africa, collecting specimens all the way; and he then entered the medical service of Southern Nigeria, where he once more saw active service on the Aro Expedition. Mr. Ansorge discovered in all nearly three hundred species of animals new to science; about half of these were vertebrates and the other invertebrates. He married in 1881, and had two sons and a daughter.

